# Effect of Treatment of Gestational Diabetes Mellitus: A Systematic Review and Meta-Analysis

**DOI:** 10.1371/journal.pone.0092485

**Published:** 2014-03-21

**Authors:** Nalinee Poolsup, Naeti Suksomboon, Muhammad Amin

**Affiliations:** 1 Department of Pharmacy, Faculty of Pharmacy, Silpakorn University, Nakhon-Pathom, Thailand; 2 Department of Pharmacy, Faculty of Pharmacy, Mahidol University, Bangkok, Thailand; Endocrine Research Center (Firouzgar), Institute of Endocrinology and Metabolism, Iran (Islamic Republic of)

## Abstract

**Objective:**

To assess the efficacy and safety of treating pregnant women with gestational diabetes mellitus in comparison to usual antenatal care.

**Methods:**

A systematic review and meta-analysis was conducted by including randomized controlled trials comparing any form of therapeutic intervention in comparison to usual antenatal care. A literature search was conducted using electronic databases together with a hand search of relevant journals and conference proceedings.

**Results:**

Ten studies involving 3,881 patients contributed to meta-analysis. Our results indicated that gestational diabetes mellitus treatment significantly reduced the risk for macrosomia (RR, 0.47; 95% CI, 0.38–0.57), large for gestational age births (RR, 0.55; 95% CI, 0.45–0.67), shoulder dystocia (RR, 0.42; 95% CI, 0.23–0.77) and gestational hypertension (RR, 0.68; 95% CI, 0.53–0.87) without causing any significant increase in the risk for small for gestational age babies. However, no significant difference was observed between the two groups regarding perinatal/neonatal mortality, neonatal hypoglycemia, birth trauma, preterm births, pre-eclampsia, caesarean section and labor induction.

**Conclusion:**

Treating GDM reduces risk for many important adverse pregnancy outcomes and its association with any harm seems unlikely.

## Introduction

Gestational diabetes mellitus (GDM) is defined as the diabetes diagnosed during pregnancy that is not clearly overt diabetes [1]. According to this definition, glycemic levels meeting the thresholds of overt diabetes are considered to have pre-existing diabetes and the rest are given diagnosis of GDM. There was much controversy in the past about association of mild hyperglycemia with adverse pregnancy outcomes, which was resolved by the landmark observational study, Hyperglycemia and Adverse Pregnancy Outcomes (HAPO) [2]. It is now established that mildly increased glucose levels during pregnancy affects both mother and fetus. It increases the incidence of macrosomia and large for gestational age (LGA) babies which in turn increases the risk for shoulder dystocia, birth trauma and caesarean section [2]–[5]. Besides, the risks for perinatal mortality, neonatal hypoglycemia, hyperbilirubinemia, gestational hypertension and pre-eclampsia also increase in GDM patients [2], [4], [6]. Chances for development of type 2 diabetes mellitus are also reported to increase in both mother and infant later in their life [6]–[8].

As the HAPO study indicated a continuous relationship between mild hyperglycemia and adverse pregnancy outcomes therefore a threshold glycemic level cannot be defined to avoid the risk completely. Consequently, confusion exists whether treatment of GDM is effective and what level of glycemic control shall produce beneficial results without increasing harm. In addition, it is not clear at what level of hyperglycemia pharmacological treatment should be initiated. Although National Institute for Health & Clinical Excellence (NICE) and American College of Obstetricians and Gynecologists (ACOG) clinical practice guidelines recommend nutritional counseling as the first line therapeutic option but American Diabetes Association (ADA) has not yet provided any specific recommendation for GDM management [1], [9], [10]. All the three professional organizations also have different diagnostic criteria which mean that the degree of hyperglycemia and risk for adverse pregnancy outcomes may also be different in patients diagnosed by these three different guideline recommendations. As there is no uniform standard to define the glucose intolerance during pregnancy, therefore, studies conducted on the topic produced varying results for different outcomes of pregnancy. This raises another question as risk for which of the adverse pregnancy outcomes can be reduced more by treating GDM. Recently, two reviews have summarized the evidence on the topic [11], [12]. In both studies, one of the important adverse pregnancy outcomes, gestational hypertension has not been analyzed explicitly and focus remained on pre-eclampsia and on combined outcome of hypertensive disorders in pregnancy. Besides, some important randomized controlled trails (RCTs) on the topic which also includes a new study published in 2013 have not been included in analysis. In view of the perplexity in this important area of health care, we conducted a systematic review and meta-analysis to precisely determine possible benefits and harms of any specific form of GDM treatment in comparison to usual antenatal care.

## Methods

We conducted this study according to Cochrane Handbook for Systematic Reviews of Interventions [13]. The data was presented according to the recommendations of the PRISMA statement [14].

### Literature search

We conducted a detailed search using electronic databases: Medline (PubMed), Cochrane Central Register of Controlled Trials (CENTRAL), http://clinicaltrials.gov register, http://clinicaltrialsresults.gov register, Cumulative Index to Nursing and Allied Health Literature (CINAHL), Latin American and Caribbean Health Sciences Literature (LILACS), Scopus and Web of Science for reports of RCTs published from respective inception up to October 2013 without any language restriction. Besides, hand search for journals and conference proceedings was also conducted. MeSH terms used were gestational diabetes, randomized controlled trial, and pregnancy. Text terms used were macrosomia, large for gestational age, and medical nutrition therapy.

### Study selection

To be eligible for this review, a study had to be a) randomized controlled trial of any specific form of therapeutic intervention used in the treatment of GDM comparing its efficacy and/ or safety to usual antenatal care, b) reporting at least one outcome of interest. Interventions included any form of treatment ranging from dietary intervention to drug therapy including insulin and antidiabetic agents administered on top of routine antenatal care. Studies including women with pre-existing diabetes mellitus were considered ineligible for this review.

### Outcomes of Interest

Outcomes were categorized into perinatal/ neonatal outcomes and maternal outcomes. In the first category, macrosomia, LGA births, shoulder dystocia, birth trauma, perinatal/ neonatal mortality, neonatal hypoglycemia, preterm birth and small for gestational age (SGA) babies were classified as outcomes of interest. In the second category, outcomes of interest included caesarean section, pre-eclampsia, gestational hypertension and labor induction.

### Data extraction and quality assessment

Data extraction and assessment of risk of bias were performed independently by two reviewers (NP, MA) using a standardized form. Inconsistencies were resolved by discussion. Data extracted from the studies included characteristics of trial patients (age, BMI, population source), interventions used, diagnostic criteria, outcome measures. Risk of bias in individual studies was assessed using the Cochrane risk of bias tool. Each study was evaluated by considering the domains of sequence generation, allocation concealment, blinding of participants/ personnel and outcome assessors, incomplete outcome data, selective outcome reporting and other risk of bias. Studies were classified as at low, uncertain and high risk of bias according to the criteria defined in the Cochrane Handbook for Systematic Reviews of Interventions [13].

### Statistical Analysis

Statistical analysis was carried out using Review Manager Software (Rev Man 5.2). Data from individual studies was combined using fixed effects meta-analysis model if heterogeneity was non-significant and random effects model was used in case significant heterogeneity was detected. Effect measure used to present the result was risk ratio (RR) with 95% confidence interval (CI). Heterogeneity between the studies was assessed using the I^2^ and Chi^2^ statistics. Heterogeneity was regarded substantial if the I^2^ is greater than 50% or there is a low P value (less than 0.10) in the Chi^2^ test for heterogeneity [13]. Publication bias was evaluated by using funnel plot and Eggers' test if five or more than five studies were included for a particular outcome [15].

## Results

**Figure 1:**
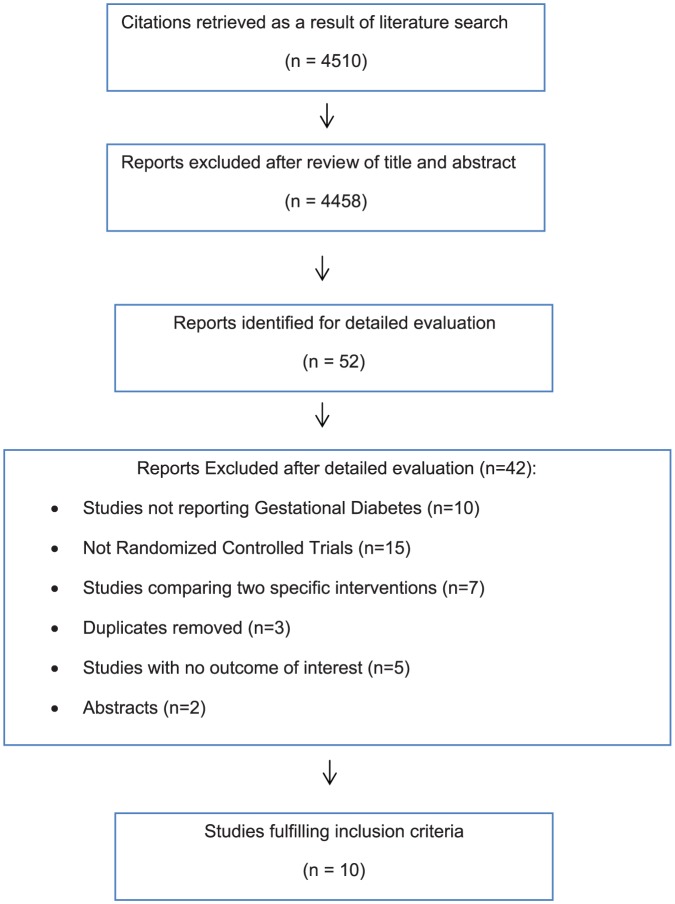
Flow chart of article selection

Study selection process is shown in Figure 1. As a result of our literature search, 4510 studies were identified initially. Titles and abstracts of these studies were reviewed and 52 relevant articles were further screened. All these studies were thoroughly investigated and at the end 10 studies met the inclusion criteria involving 3881 participants [16]–[25]. Characteristics of these studies are presented in Table 1. In these studies, interventions used in the experimental group were dietary modification and glucose monitoring; insulin was administered mostly if glycemic targets were not met with dietary modification. These interventions were compared to routine antenatal care in the control group.

**Table 1:** Characteristics of the included studies

Study, Country of Origin	N	Mean Age (SD)	Mean BMI (SD)	Intervention		Diagnostic Criteria	Patients on Insulin^Ψ^
				Exp.	Control		
Bevier 1999[16], USA	83	26.8 (5.7)	NA	Diet/Insulin	Usual care, Insulin if RBG >120 mg/dl	50 g GCT 1 h>140 mg/dl & negative on 100 g OGTT	3%
Bonomo 2005[17], Italy	300	30.9 (4.9)	23 (4.4)	Diet	Usual care	50 g GCT 1 h≥140 mg/dl & negative on 100 g OGTT	NA
Crowther 2005[18], Australia/UK	1000	30.5 (5.5)	26.4	Diet/Insulin	Usual care	75 g OGTT 2 h≥140 mg/dl ≤198 mg/dl	20%
Deveer 2013[19], Turkey	100	30.3 (5.7)	28.5 (4.2)	Diet	Usual care	50 g GCT 1 h≥140 mg/dl<180 mg/dl & negative on 100 g OGTT	NA
Garner 1997[20], Canada	300	30.7 (4.7)	NA	Diet/Insulin	Usual care. Insulin if FPG>140 mg/dl	75 g OGTT Any abnormal: F>86.4 mg/dl, 1 h>196 mg/dl, 2 h>172 mg/dl	24%
Landon 2009[21], USA	958	29.1 (5.7)	30 (5)	Diet/Insulin	Usual care	100 g OGTT F<95 mg/dl& 2 abnormal: 1 h≥180 mg/dl, 2 h≥155 mg/dl, 3 h≥140 mg/dl	7.6%
Langer 1989[22], USA	126	29.5 (5.5)	NA*	Diet/Insulin	Usual care	100 g OGTT 2 Abnormal: F≥105 mg/dl, 1 h≥190 mg/dl, 2 h≥165 mg/dl, 3 h≥145 mg/dl	35%
Li 1987[23], Hong Kong	158	28.3 (4.5)	NA	Diet	Usual care	100 g OGTT 2 abnormal: F≥105 mg/dl, 1 h≥190 mg/dl, 2 h≥165 mg/dl, 3 h≥145 mg/dl	NA
O'Sullivan 1966[24], USA	615	30.8 (NA)	NA	Diet & Insulin for all	Usual care	100 g OGTT 2 Abnormal: F≥110 mg/dl, 1 h≥170 mg/dl, 2 h≥120 mg/dl, 3 h≥110 mg/dl	100%
O'Sullivan 1974[25], USA	241	30(NA)	NA	Diet &Insulin for all	Usual care	100 g OGTT 2 Abnormal: F≥90 mg/dl, 1 h≥165 mg/dl, 2 h≥145 mg/dl, 3 h≥125 mg/dl	100%

Exp: Experimental. ^Ψ^: Percentage of patients requiring insulin therapy in experimental group. BMI: Body mass index. SD: Standard deviation. NA: Not available. GCT: Glucose challenge test. OGTT: Oral glucose tolerance test, RBG: Random blood glucose, FPG: Fasting plasma glucose.*BMI was ≥27 in 38% of patients in experimental group and 41% of patients in control.

### Risk of Bias in the Included Studies

**Figure 2:**
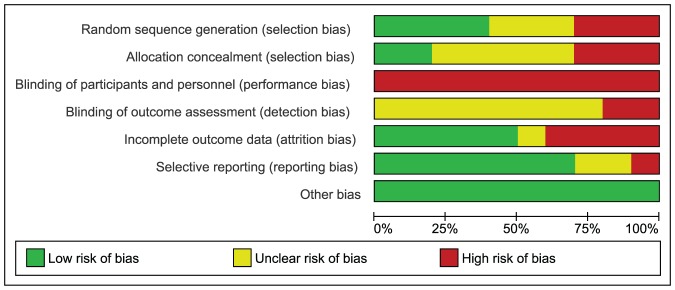
Risk of bias graph

**Figure 3:**
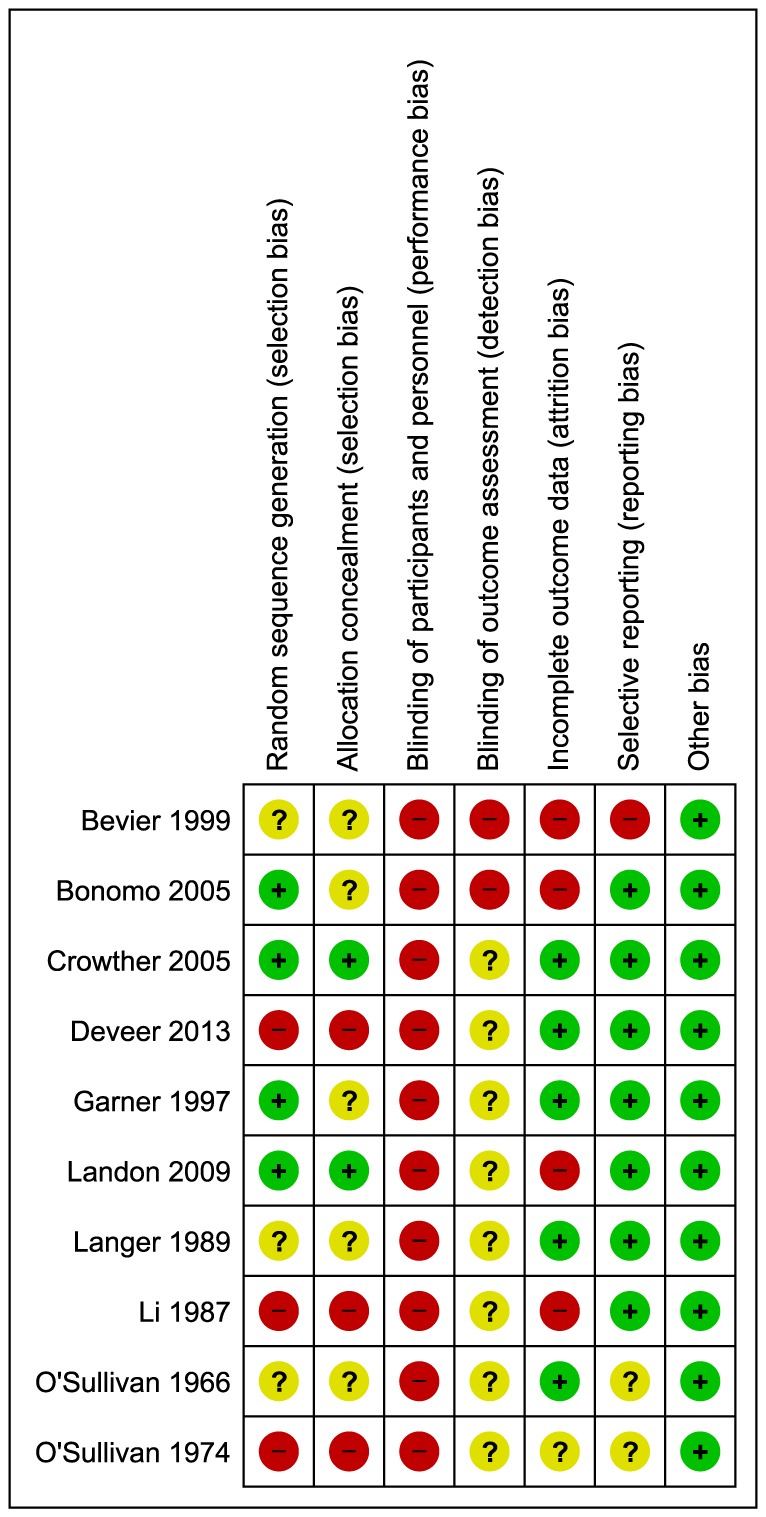
Risk of bias summary

Of the ten studies included in the review, sequence generation was performed and described adequately in four studies only [17], [18], [20], [21]. Three studies were at high risk of bias for sequence generation as it was based on the days of week in one study [19],and in two studies, alternation was performed [23]. For the rest of studies methods adopted for sequence generation remained unclear [16], [17], [22], [24]. Allocation was properly concealed in two studies only [18], [21]. Three studies were at high risk of bias for this domain as allocation was based on alternation [19], [23], [25],and for the rest of studies it remained unclear. No study was double blinded and, therefore, remained at high risk of performance bias. Blinding of outcome assessors was at high risk of bias in two studies [16], [17], whereas in remaining studies, it remained unclear. However, double blinding was avoided mostly due to the reason that blinding was neither practical nor ethical due to multiple interventions used in the study and potential hazards associated with the disease condition. Four trials had incomplete outcome data and patients were either excluded or lost to follow up in both the intervention and the control groups [16], [17], [21], [23]. Among the remaining six studies, five were at low risk of attrition bias [18]–[20], [22], [24], and in one study it remained unclear [25]. Reporting bias was detected in one study only [16], while in two studies incomplete information was provided for this domain [24], [25]. In the study at high risk of reporting bias, definition and incidence of macrosomia was not clear. In other two studies at unclear risk of reporting bias, many outcomes of potential importance were ignored and it remained unclear what study protocol had actually conceived. There was no other apparent risk of bias in all included studies. Fig 2 and 3 shows risk of bias summary.

### Perinatal/ Neonatal Outcomes

Macrosomia and LGA births were reported as outcomes of interest in the majority of studies. Mostly macrosomia was defined as birth weight equal to or above than 4000 g. However, O'Sullivan et al. [24], defined macrosomia as birth weight above 4100 g, while Bevier et al.[16] did not define macrosomia properly and a combined result of macrosomia and LGA births was provided. Garner et al.[20], defined macrosomia as birth weight above 4500 g but data was also provided seperately for birth weight above 4000 g. To keep uniformity with other studies we included the later statistics in our analysis of macrosomic infants. Likewise, LGA birth was defined as birth weight above 90^th^ percentile for gestational ageand SGA was defined as birth weight below 10^th^ percentile for gestational age in majority of studies.

**Figure 4:**
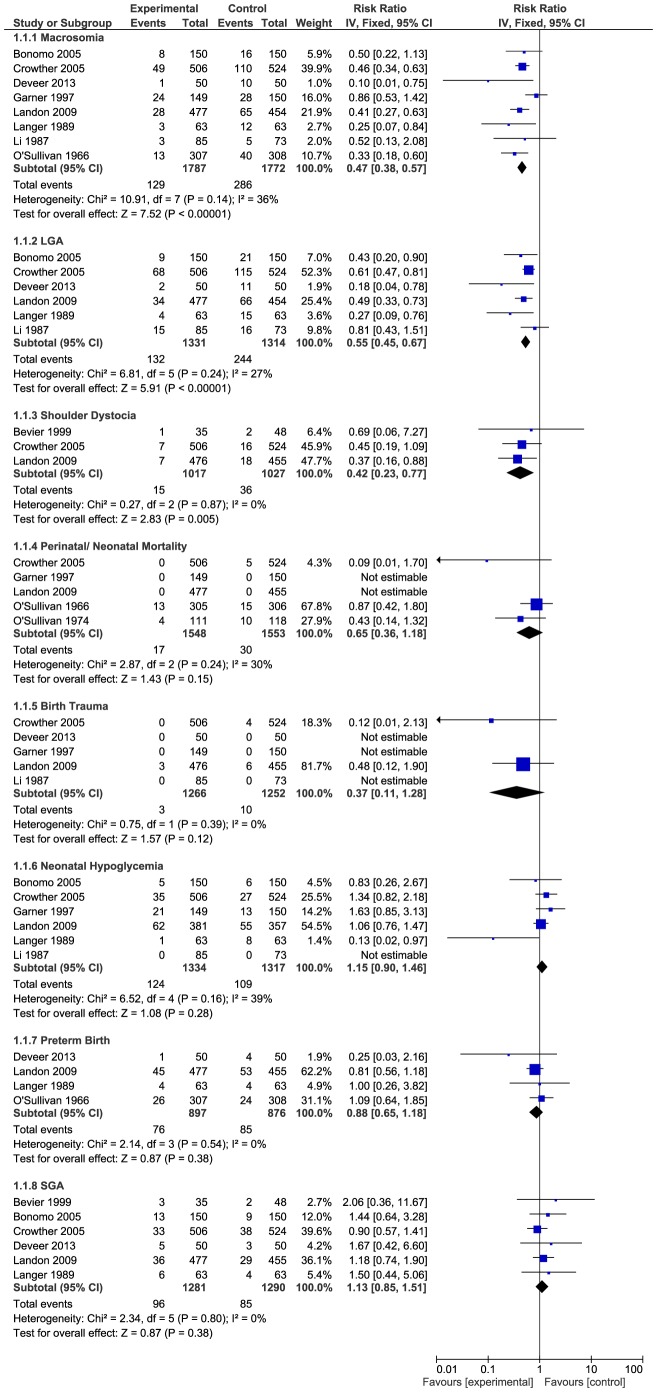
Effects of treatment on perinatal / neonatal outcomes

In our review, infants born to GDM mothers in the treatment group were at significantly lower risk for macrosomia (RR, 0.47; 95% CI, 0.38–0.57, p-value<0.00001), LGA birth (RR, 0.55; 95% CI, 0.45–0.67, p-value<0.00001) and shoulder dystocia (RR, 0.42; 95% CI, 0.23–0.77, p-value = 0.005) as compared to routine care group (Fig 4). Perinatal/neonatal mortality was observed mostly in the two older stuides of O'Sullivan et al.[24], [25], while in the recent studies very few such deaths were reported. Nonetheless, risk for perinatal/ neonatal mortality was lower, but non-significantly so, in the treatment group as compared to control (RR, 0.65; 95% CI, 0.36–1.18). Similarly, infants born to GDM mothers in the treatment group were non-significantly at a lower risk for birth trauma (RR, 0.37; 95% CI, 0.11–1.28) and preterm births (RR, 0.88; 95% CI, 0.65–1.18) (Fig 4). The risk for neonatal hypoglycemia slightly but non-significantly increased in the intervention group (RR, 1.15; 95% CI, 0.90–1.46). There was no significant increase in the risk for SGA babies (RR, 1.13; 95% CI, 0.85–1.51) in the intervention group as compared to usual care (Fig 4). No significant heterogeniety was detected across all studies for all outcomes of interest. We evaluated publication bias for macrosomia, LGA births, perinatal/neaonatal mortality,neonatal hypoglycemia and SGA births. Publication bias was detected for SGA births only (Egger's test: intercept, 1.13121; 95% CI, 0.06779–2.19463, p-value = 0.04183). We observed a minor impact on the effect estimate of SGA births after making adjustments for publication bias by using trim and fill method (RR, 1.07; 95% CI, 0.82–1.40).

### Maternal Outcomes

**Figure 5:**
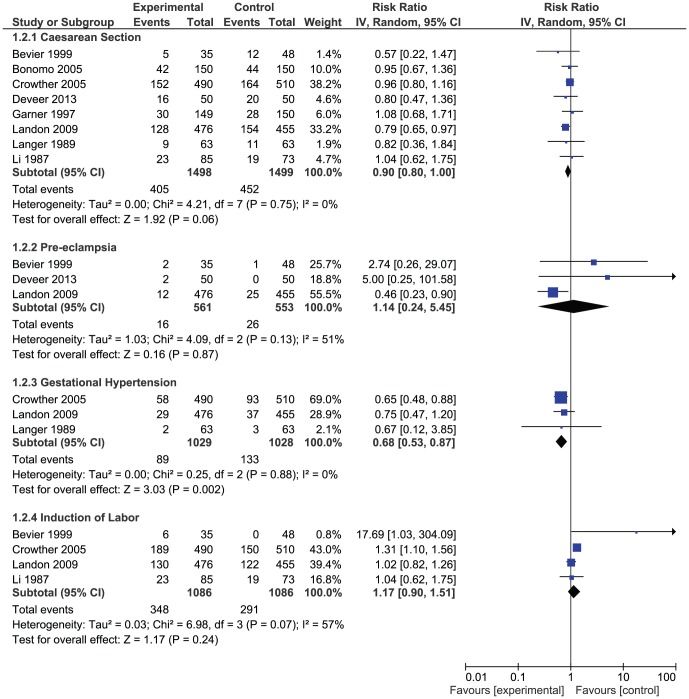
Effects of treatment on maternal outcomes

The risk for caesarean section (RR, 0.90; 95% CI, 0.80–1.00) was less likely in the intervention group, but statistical significance was not achieved (Fig 5). However, the risk of gestational hypertension was reduced by 32% (95% CI, 13%–47%, p-value = 0.002) with the intervention (Fig 5). We observed a non-significant increase in the risk for pre-eclampsia (RR, 1.14; 95% CI, 0.24–5.45) and labor induction (RR 1.17; 95% CI, 0.90–1.51), in the intervention arm. Heterogeniety was non-significant for caesarean section and gestational hypertension whereas significant heterogeniety was observed for pre-eclampsia and labor induction.Publication bias was evaluated for caesarean section. No publication bias was detected for this outcome.

## Discussion

Treatment of GDM remained controversial mostly due to the lack of a uniform standard for defining glucose intolerance during pregnancy [26]. Because of this reason, individual studies on the topic produced varying results causing confusion about efficacy and safety of GDM treatment. We found dietary intervention along with glucose monitoring as the primary therapeutic choice in all the studies meeting our inclusion criteria and insulin therapy was initiated if dietary modification failed to control glycemic levels. Based on the data from these studies, results of our analysis indicated that treating GDM women with a specific therapeutic intervention decreases incidence of many adverse pregnancy outcomes. We observed significant decrease in the risk of macrosomia, LGA births and shoulder dystocia in infants. Reduction in the risk of macrosomic and LGA babies is likely to have important implications for the infants in the long term also as these outcomes have been linked with delayed motor development, premenopausal breast cancer, obesity and diabetes later in life [8], [27]–[30]. Results remained non-significant for perinatal/neonatal mortality, birth trauma, preterm births and neonatal hypoglycemia and any likely benefit of treatment on these outcomes was not observed. However, of potential importance was our observation that much of the incidence of neonatal mortality was noticed in the two older trials of O'Sullivan et al.[24], [25]conducted in USA, while only five such cases were reported in the control group by Crowther et al.[18] in a recent study conducted in Australia and UK. This may be due to improvements in antenatal care in developed world. If such is the case, then these findings may have profound implications for GDM patients in developing countries with fragile health care systems. Nonetheless, studies with a large sample size are required, ideally conducted in developing countries, to confirm these findings. One of the safety concerns of GDM treatment is increased risk for SGA babies if strict glycemic goals are achieved [31]. Our analysis indicated that GDM treatment is not associated with such an outcome.

Suspected macrosomia is reported to increase risk of caesarean section and labor induction [32]. Although risk of macrosomia was decreased significantly in our analysis however, risk of caesarean section showed a non-significant reduction in the treatment group as compared to control. Similarly, we noticed that GDM treatment had no significant impact on the risk of induced labor. However, we consider these both outcomes being at high risk of performance and detection bias as none of the studies was double blinded and knowledge of treatment allocation might have influenced the decisions of health care providers involved in the study. On the other hand, we noticed a significant reduction in the incidence of gestational hypertension in the treatment group. Decrease in the rate of gestational hypertension is likely to reduce the incidence of pre-eclampsia and caesarean section due to its relationship with the rate of these pregnancy outcomes [33], [34]. Nonetheless, our results remained non-significant for both caesarean section and pre-eclampsia.

Our results for majority of outcomes of interest which includes macrosomia, LGA births and shoulder dystocia are in line with the earlier conducted reviews of Falavigna et al. [11] and Hartling et al. [12]. However, there are some disagreements in interpreting results from individual studies. We did not include data on macrosomia and LGA births from Bevier et al. study [16] due to ambiguity in rate and definition of these outcomes, whereas Hartling et al. [12] ignored this aspect while analyzing both outcomes. Besides, both of these reviews reported a significantly lower risk of pre-eclampsia in the intervention group, while our analysis showed non-significant results on this outcome. This may be due to the reason that the definition of pre-eclampsia in Crowther et al. [18] study was more relevant to gestational hypertension instead of pre-eclampsia. Therefore, we considered it more appropriate to include the data on pre-eclampsia from Crowther et al. [18] study into gestational hypertension. Both reviews provided results for a combined outcome of hypertensive disorders in pregnancy without explicitly reporting on outcome of gestational hypertension [11], [12]. We for the first time reported that GDM treatment also reduces the risk for gestational hypertension in addition to many other adverse outcomes.

Looking at individual trials, we observed that glycemic control is difficult to achieve with dietary intervention in patients with a more severe form of disease as reflected from the diagnostic criteria employed in the study and percentage of patients requiring insulin therapy [18], [22]. This means that individual patient characteristics play an important role in achieving glycemic goals with a specific intervention. Nonetheless, we noticed that the current glycemic targets defined for GDM management can easily be achieved with nutrition therapy in majority of patients. Mostly, studies conducted on the topic have targeted nearly same glycemic levels and produced beneficial results without increasing harm. Current recommendations for target glycemic levels in GDM patients are <96 mg/dl at fasting, <140 mg/dl at 1 hour postprandial and <120 mg/dl at 2 hours postprandial [35].Mean capillary glucose levels <87 mg/dl are considered to be associated with increased incidence of SGA babies [35]. Nevertheless, a recent pooled analysis of plasma glucose levels in non-diabetic pregnant women indicated much lower levels as compared to current recommendations. These estimates are 71±8 mg/dl at fasting, 109±13 mg/dl at 1 hour postprandial and 99±10 mg/dl at 2 hour postprandial [36]. It is not known whether further lowering of target glucose levels will further improve the outcomes of therapy without raising any safety concern. However, this is a new area of research that needs to be explored further.

### Strengths and Limitations

This is the largest and the most updated review of RCTs conducted on the topic till date. Therefore our results are more precise than the earlier conducted reviews [11], [12].In addition, studies were consistent across all the outcomes of interest and no statistically significant heterogeneity was detected except for labor induction, despite the studies were conducted over a large span of time. We detected publication bias for SGA births only and after making adjustment by trim and fill method no substantial impact on effect estimate was observed. Nonetheless, we could not achieve sufficient power to report significant results for some important outcomes like perinatal/ neonatal mortality, preterm birth, SGA infants, caesarean section and pre-eclampsia. Lack of blinding in the studies is thought to affect validity of data, especially for an objective outcome like caesarean section and labor induction. Most of the studies included in the review were of small sample size and results were dominated by two large studies of Crowther et al.[18] and Landon et al.[21]. Furthermore, these two studies and other ones were conducted mostly in developed world therefore extrapolation of these results to a population residing in developing countries is difficult due to differences in life style, health care facilities and ethnicities. Besides, there was a large variation in the diagnostic criteria among studies implying that patients with different degrees of hyperglycemia were involved.

## Conclusion

Our results provided robust evidence in favor of GDM treatment which comprised of dietary modification and glucose monitoring along with insulin supplements, if required. This intervention is likely to reduce the risk for several key outcomes of interest like macrosomia, LGA births, shoulder dystocia and gestational hypertension. In addition to short term benefits, reduction in incidence of these outcomes is expected to produce long term benefits both for the infant and mother. Our analysis also indicated that treatment of GDM using above mentioned intervention is not associated with any potential harm including SGA births. However, further studies of large sample size are required to be conducted in both developed and developing countries to determine the impact of treatment on many important low incidence adverse pregnancy outcomes.
